# Loss of Expression and Promoter Methylation of SLIT2 Are Associated with Sessile Serrated Adenoma Formation

**DOI:** 10.1371/journal.pgen.1003488

**Published:** 2013-05-09

**Authors:** Andrew D. Beggs, Angela Jones, Neil Shepherd, Abed Arnaout, Caroline Finlayson, A. Muti Abulafi, Dion G. Morton, Glenn M. Matthews, Shirley V. Hodgson, Ian P. M. Tomlinson

**Affiliations:** 1 Molecular and Population Genetics Laboratory, Wellcome Trust Centre for Human Genetics, University of Oxford, Oxford, United Kingdom; 2 Department of Surgery, Croydon University Hospital, Croydon, United Kingdom; 3 Department of Surgery, School of Cancer Sciences, University of Birmingham, Birmingham, United Kingdom; 4 Department of Histopathology, Cheltenham General Hospital, Cheltenham, United Kingdom; 5 Department of Histopathology, Croydon University Hospital, Croydon, United Kingdom; 6 Department of Histopathology, St. George's Hospital, London, United Kingdom; 7 Epithelial Research Group, School of Cancer Sciences, University of Birmingham, Birmingham, United Kingdom; 8 Department of Cancer Genetics, St. George's University of London, London, United Kingdom; University of Washington, United States of America

## Abstract

Serrated adenomas form a distinct subtype of colorectal pre-malignant lesions that may progress to malignancy along a different molecular pathway than the conventional adenoma-carcinoma pathway. Previous studies have hypothesised that BRAF mutation and promoter hypermethylation plays a role, but the evidence for this is not robust. We aimed to carry out a whole-genome loss of heterozygosity analysis, followed by targeted promoter methylation and expression analysis to identify potential pathways in serrated adenomas. An initial panel of 9 sessile serrated adenomas (SSA) and one TSA were analysed using Illumina Goldengate HumanLinkage panel arrays to ascertain regions of loss of heterozygosity. This was verified via molecular inversion probe analysis and microsatellite analysis of a further 32 samples. Methylation analysis of genes of interest was carried out using methylation specific PCR (verified by pyrosequencing) and immunohistochemistry used to correlate loss of expression of genes of interest. All experiments used adenoma samples and normal tissue samples as control. SSA samples were found on whole-genome analysis to have consistent loss of heterozygosity at 4p15.1–4p15.31, which was not found in the sole TSA, adenomas, or normal tissues. Genes of interest in this region were PDCH7 and SLIT2, and combined MSP/IHC analysis of these genes revealed significant loss of SLIT2 expression associated with promoter methylation of SLIT2. Loss of expression of SLIT2 by promoter hypermethylation and loss of heterozygosity events is significantly associated with serrated adenoma development, and SLIT2 may represent a epimutated tumour suppressor gene according to the Knudson “two hit” hypothesis.

## Author Summary

Polyps in the bowel are the precursors of development of bowel cancer. Much is known about the biology and pathology of these polyps. These polyps have classically been divided into two main types: adenomas and hyperplastic polyps. The former has pre-malignant potential, whereas it is thought the second does not. Within the last 10 years, a third type of polyp has been discovered, called the “serrated adenoma,” that has features of both adenomas and hyperplastic polyps. However, they seem to have very different characteristics, and we are not certain of the distinct molecular event that leads to their development. In this study we investigate the genetic differences that set apart serrated adenomas from other types of polyps. We report on a gene change within these polyps that seems to be associated with their development. The gene change is highly unusual as it consists of the loss of one copy of the gene as well as methylation of the other gene. This methylation switches off the remaining copy of the gene and stops its associated protein from being expressed in the polyp. To our knowledge, this is the first time this phenomenon has been seen.

## Introduction

Serrated adenomas are a type of colorectal polyp [1] that possess features of both hyperplastic and adenomatous polyps. The classical feature of serrated adenomas is saw-toothed infolding of dysplastic colonic epithelium as described by Longacre et al (2). The classification of serrated adenomas into distinct types has presented a challenge. Snover et al [2] suggested subdivision into sessile serrated adenoma (SSA), traditional serrated adenoma (TSA), mixed serrated polyp (MSA) and sessile serrated polyp (SSP), with the latter two subtypes being reserved for difficult to classify or atypical mixed polyps with serrated morphology. Rex et al [3] suggested polyps be subdivided into SSA/P (with or without cytological dysplasia), TSA and stated that these two lesions were the predominant pre-malignant lesions in the serrated pathway. They also stated that dysplasia observed in SSA/P represented the progression of a SSA towards malignancy. The differences between SSAs and TSAs were examined by Torlakovic et al [4] who described SSAs as flat, sessile lesions with crypts that grow parallel to the muscularis mucosae and highly variable and irregular Ki67 expression, whereas TSAs are pedunculated lesions with similar crypts to SSA but low Ki67 expression and a highly eosinophillic cytoplasm. SSA are thought to form a unique subset of colorectal polyps with a distinct molecular pathogenic pathway, known as the “serrated pathway”.

The hypothesised molecular pathway in the development and progression of SSA is thought to be different from the conventional adenoma-carcinoma pathway. The most important molecular event known to be highly frequent in the pathogenesis of SSAs is an activating mutation in *BRAF* (1). Multiple studies have found *BRAF* mutations within SSAs at frequencies of 62–100% [5]–[8]. However, *BRAF* mutations are not specific to serrated tumours, also being found in colonic adenomas and cancers without a serrated morphology [9]. Also, BRAF mutations have been found in TSA with dysplasia at a high frequency [10]. Consequently *BRAF* mutations in isolation cannot be said to cause sessile serrated morphology. It has been proposed that a hyperplastic polyp (HP) is initiated by a *BRAF* mutation. The HP then acquires the CpG island methylator (CIMP) phenotype, leading to silencing of genes such as *IGFBP7* and *MLH1*. This is accompanied or followed by progression to a SSA. Methylation of the *MLH1* promoter is also hypothesised to be responsible in part for the acquisition of microsatellite instability and for progression to microsatellite-unstable cancer. However, a set of *BRAF*-mutant, CIMP-high, microsatellite stable cancers also exists [1] and the origins of this type of tumour are currently unknown.

Since the serrated adenoma molecular pathway is not fully elucidated, and is unknown whether a common pathway exists to give the serrated phenotype, which then leads to the formation of SSA or TSA, we used genome-wide SNP typing to screen for regions of copy number change and loss of heterozygosity in a set of sessile serrated adenomas. On the basis of our results, we investigated candidate genes in regions of loss of heterozygosity for promoter methylation and changes in expression.

## Results

Of the 42 total serrated adenomas, 20/42 were from male patients, and 22/42 from female patients. The median age of patients was 69 years (range 34–87 years). In terms of location, 8/42 were located in the caecum, 2/42 in the ascending colon, 11/42 in the sigmoid colon and 21/42 in the rectum. Of the forty two serrated adenomas, 1/42 was a mixed traditional serrated & sessile serrated adenoma, 1/42 was a mixed sessile serrated/tubovillous adenoma (reported predominantly as a sessile serrated adenoma), 3/42 were filiform TSA [3] and the remaining 37/42 were sessile serrated adenomas. In terms of levels of dysplasia, the mixed SSA/Adenoma was mildly dysplastic, and of the 37 SSA, 19 had mild dysplasia and 18 had no dysplasia. We divided the SA's into two sets, an initial discovery set consisting of 10 SA's (9 SSA, 1 TSA) and a second verification set consisting of 32 SA's (2 TSA, 1 mixed SSA, 29 SSA).

In the classical adenoma samples, 6/10 were from female patients and 4/10 from male patients, with a median age of 58 years (range 45–73 years). They were located in the sigmoid colon (2/10), Caecum (3/10) and Descending colon (2/10) and rectum (3/10). In the classical adenoma tissue microarray there were 100 samples, of which 45/100 were from male patients and 55/100 from female patients with a median age of 58 years (range 30–85 years). In terms of histology, 55/100 were mildly dysplastic, 40/100 moderately dysplastic and 5/100 severely dysplastic. Of the 100 polyps on the array, 22/100 were located in the rectum, 25/100 in the sigmoid, 10/100 in the descending colon, 21/100 in the transverse colon, 10/100 in the ascending colon and 12/100 in the caecum.

**Figure 1:**
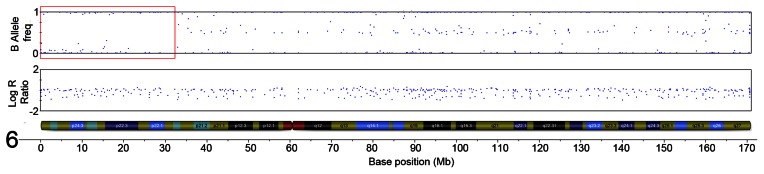
Region of CN-LoH in Chromosome 6 in sample SA2 (specific area illustrated by red box). The plot below demonstrates a loss of heterozygosity in this region shown by the loss of heterozygous SNPs with a B allele frequency of ∼0.5, with only homozygous SNPs remaining. This event is copy-neutral because there is no change in the Log R ratio seen in the corresponding plot.

**Figure 2:**
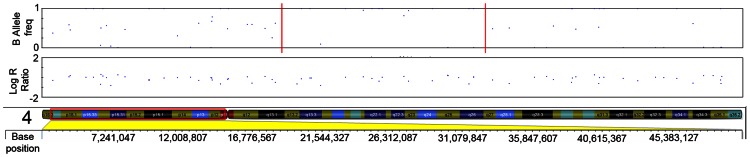
LRR and BAF plot of region of CN-LOH within serrated adenomas. Red dotted line in B-allele frequency plot indicates region of CN-LOH; Red box indicates chromosomal position within ideogram.

The initial ten serrated adenoma samples (9 SSA, 1 TSA) from the discovery set were successfully genotyped on the Goldengate array. Of the 9 SSA, 4 had dysplasia and 5 did not. The average genotyping call rate was 0.993. Samples were assessed for copy number changes and LoH events using the log R ratio and the B allele frequency in Illumina GenomeStudio software. There were no large-scale chromosomal gains or losses, except for one sample where there was copy-neutral LoH for the majority of the p-arm of chromosome 6 (Figure 1). There was only one recurrent region of small-scale copy number change (Figure 2), a single-copy deletion at chromosome 4p15.1–4p15.31 (Chr4:19,837,583–33,138,129). Nine out of ten samples (all SSA samples, with and without dysplasia) demonstrated this region of copy neutral LoH and this result was confirmed by MIP array analysis in the single SSA sample for which the assay was successful. In total 2 SSA samples were analysed via MIP array with 1 sample failing due to insufficient sample quality. Due to cost constraints the whole genome genotyping was not repeated on the subsequent 32 samples of the verification set. The sole SA in the discovery set which GoldenGate array analysis did not demonstrate LoH at 4p15.1–15.31 was the SA classified as a TSA.

In order to validate the SNP array data, microsatellite LOH analysis was performed on the 9 initial SSA samples (SA A - I) in the discovery set (specifically excluding the single TSA sample). The results of this are shown in Table 1, as well as the measured heterozygosity at these loci, the genetic and cytogenetic position and BP length of the microsatellite. Although there was variable performance of markers and hence LOH frequency, presumably related to chance differences in allelic amplification from FFPE-derived DNA, LOH in the region was confirmed. Although there was no normal tissue we noted that all SSA samples appeared to be homozygous. We could not distinguish this between acquired and constitutional homozygosity however the initial samples matched with the SNP arrays so we assume this represented acquired homozygosity. In the sessile serrated adenoma samples of the discovery set, AFM112XD4 was found to show LOH in all 9 samples except sample C. Being the most informative marker in the region, AFM112XD4 was used to test LOH in the second verification set of 32 SAs. Within this group, of the 29 SSA's, 26/29 had LOH (89.7%). The mixed SSA also demonstrated LOH, whereas the 2 TSA's did not demonstrate LoH, giving a total of twenty-seven of 32 (87%) samples showing LOH. This was significantly different to the observed rate of heterozygosity in the blood control samples (78% heterozygosity, p<0.001, Z = −6.930, Rank sum). This also reassured us that the observed homozygosity was highly likely to be acquired rather than constitutional. None of the 10 classical adenoma samples had LoH at this marker.

**Table 1:** Microsatellite LoH status by marker and by sample (ordered from left to right by position).

Sample	D4S230	D4S418	D4S914	D4S313	D4S616	D4S1593	D4S2289	AFM112XD4
SSA-A	No LoH	No LoH	LoH	LoH	LoH	LoH	LoH	LoH
SSA-B	LoH	LoH	LoH	LoH	No LoH	LoH	No LoH	LoH
SSA-C	No LoH	No LoH	LoH	No LoH	LoH	No LoH	No LoH	LoH
SSA-D	LoH	LoH	LoH	LoH	No LoH	LoH	LoH	LoH
SSA-E	No LoH	LoH	LoH	LoH	No LoH	LoH	LoH	LoH
SSA-F	LoH	LoH	LoH	LoH	No LoH	LoH	No LoH	LoH
SSA-G	No LoH	No LoH	LoH	LoH	LoH	LoH	LoH	LoH
SSA-H	LoH	LoH	LoH	LoH	No LoH	No LoH	No LoH	LoH
SSA-I	LoH	LoH	LoH	LoH	LoH	LoH	LoH	LoH
Total LoH	55.6%	66.7%	100%	88.9%	44.4%	77.8%	55.6%	100%
Location (BP)	chr4:28248451-28248808	chr4:28974307-28974656	chr4:30368652-30368959	chr4:27297331-27297714	chr4:23157479-23157854	chr4:20853502-20853867	chr4:19299850-19300279	chr4:30364833-30364998
Cyto band	4p15.1	4p15.1	4p15.1	4p15.2	4p15.2	4p15.31	4p15.31	4p15.1
BP Size	191–217	210–226	132–133	242–243	225–226	138–154	284	132
Genes	-	-	*PCDH7*	-	-	*KCNIP4*	-	PCDH7, SLIT2
Measured heterozygosity	56.3%	87.5%	41.0%	81.3%	83.3%	45.8%	62.5%	78.0%

In this region of LoH there are 7 genes: *SLIT2* (ENSG00000145147), *GPR125* (ENSG00000152990), *PACRGL* (ENSG00000163138), *DHX15* (ENSG00000109606), *SOD3* (ENSG00000109610), *RBPJ* (ENSG00000168214) and *PCDH7* (ENSG00000169851). Of these, we prioritised two genes – *SLIT2* and *PCDH7 –* as candidate LOH targets, based on their functions. *SLIT2* is a negative regulatory component of the DCC/netrin pathway (14) which leads to malignant transformation in colorectal epithelial cells by Hakai-mediated E-cadherin degradation [11], [12], and *PCDH7* encodes a protocadherin, the C-isoform of which interacts with ATM in double strand break repair (15).

**Figure 3:**
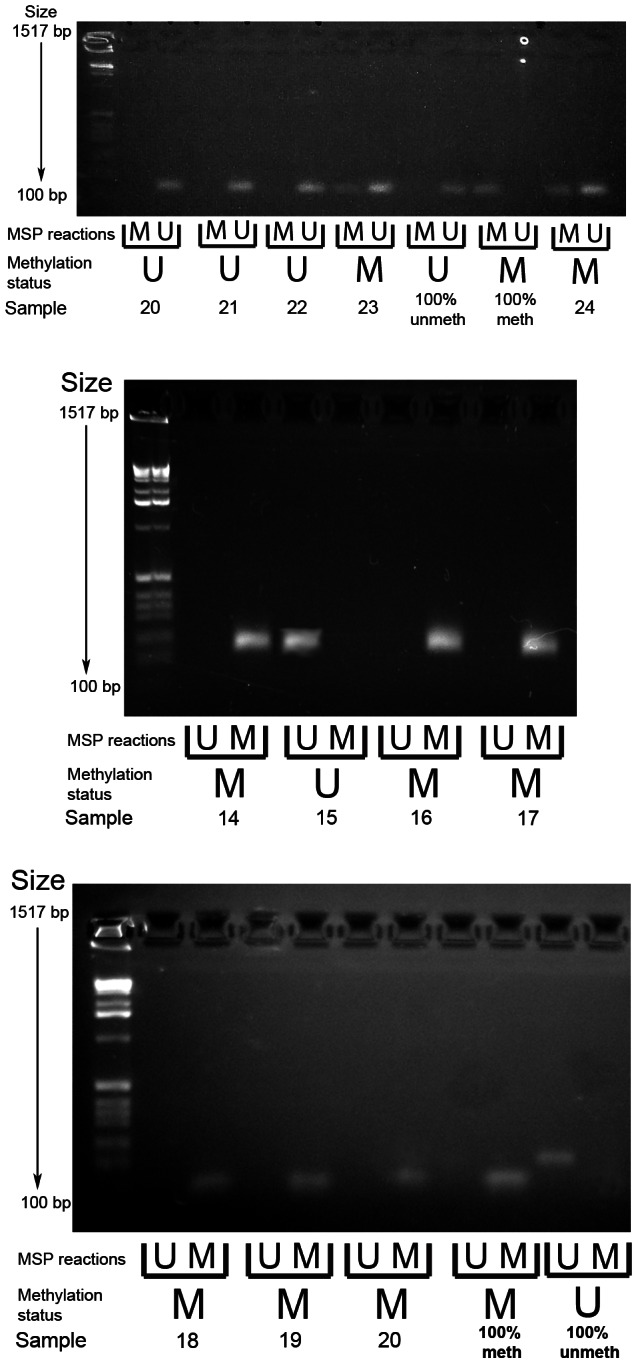
Example gel image of PCDH7 promoter methylation-specific PCR (top gel) and SLIT2 methylation-specific PCR (bottom and middle gels). MSP reactions row refers to wells for the methylated and unmethylated reactions for the methylation specific PCR. Methylation status refers to the final determination of methylation within each serrated adenoma sample. Row key: MSP reactions – M = methylation specific reaction, U = unmethylated specific reaction; Methylation status – M = methylated, U = unmethylated.

Methylation specific PCR of the *PCDH7* and *SLIT2* promoter regions was carried out in the verification group only. No further examination of other gene specific promoter regions in this area (i.e. *GPR125*,*PACRGL*,*DHX15*,*SOD3* or *RBPJ*) was carried out as a visual examination of the ENCODE DNA methylation tracks in the UCSC Genome Browser did not suggest any long range methylation in this region. For *PCDH7*, 12/32 (37.5%) SAs had methylation (12/29 SSAs, 0/1 mixed SSA/TSA, 0/2 TSA), whereas none of the classical adenoma (0/10) samples and 1/5 (20%) of the normal mucosa samples were methylated (Figure 2). For *SLIT2*, 31/32 (96.9%) SAs had methylation (29/29 SSA's, 1/1 mixed TSA/SSA, and 0/2 TSAs). There was no methylation in the adenoma (0/10) or normal mucosa samples (0/5) (Figure 3). No methylation analysis of additional normal mucosa samples was carried out at this point as the frequency of methylation observed in SLIT2/PCDH7 was so low it was thought unlikely to add any additional information. The MSP result was verified in a limited subset of samples using pyrosequencing. Five CpG dinucleotides were interrogated in the pyrosequencing PCR, based on the coverage of the MSP, and abnormal methylation was defined as a methylation percentage greater than two times the standard deviation of the average methylation in normal mucosa. It was found that in SSAs, there was consistent and informative promoter methylation at the 2^nd^, 3^rd^ and 4^th^ CpG's within the MSP region (Table 2). There was no hypermethylation in a caecal polyp, nor in normal mucosa. As part of a separate experiment conducted in the laboratory, it was found that there was hypermethylation in *SLIT2* within a panel of cell lines (Table 2). Using a separate pyrosequencing primer for SLIT2 (PM00018634) , the SSA were checked for long range methylation in the serrated adenomas further down the CpG island. There was no methylation seen (Table 2).

**Table 2:** Pyrosequencing results, with median methylation of normal tissue shown at bottom.

			SLIT2 pyrosequencing of MSP site					Long range SLIT2
Sample	Tissue type	Location	CpG1	CpG2	CpG3	CpG4	CpG5	Average methylation
Serrated adenomas	SSA/P	Caecum	22%	70% *	35% *	51% *	26% *	10%
	SSA/P	Rectum	35% *	61% *	48% *	48% *	0%	0%
	TSA	Caecum	33% *	33%	29%	0%	21% *	9%
	SSA/P	Rectum	36% *	64% *	49% *	84% *	0%	0%
100% methylated	Human control DNA	-	83% *	88% *	82% *	94% *	91% *	85%
Caecal polyp	Adenoma with low grade dysplasia	Caecum	19%	22%	15%	23%	14%	-
Normal mucosa	Normal mucosa	Ascending colon	29% *	36% *	17%	38%	18%	-
	Normal mucosa	Transverse colon	9%	15%	11%	14%	10%	-
	Normal mucosa	Left colon	16%	29%	0%	32%	17%	-
	Normal mucosa	Descending colon	16%	17%	20%	26%	9%	-
	Normal mucosa	Sigmoid	11%	14%	10%	13%	9%	-
	Normal mucosa	Rectum	14%	14%	13%	17%	8%	-
	Normal mucosa	Distal rectum	11%	19%	18%	36%	13%	-
Cell lines	C80	-	100% *	91% *	93% *	100% *	67% *	-
	HDC57	-	93% *	69% *	67% *	97% *	66% *	-
	LOVO	-	79% *	90% *	90% *	100% *	71% *	-
	C99	-	95% *	90% *	88% *	100% *	74% *	-
	LS123	-	100% *	87% *	94% *	100% *	56% *	-
	CACO2	-	96% *	91% *	94% *	100% *	68% *	-
	HDC82	-	84% *	80% *	92% *	100% *	67% *	-
	COLO741	-	46% *	52% *	67% *	73% *	46% *	-
	LS411	-	40% *	38% *	36% *	42%	23% *	-
	CCK81	-	87% *	90% *	92% *	100% *	70% *	-
	SNU175	-	64% *	60% *	80% *	96% *	64% *	-
Median	Normal tissue		16%	19%	15%	25%	13%	-
SD	Normal tissue		6%	8%	6%	9%	4%	-
2SD+median	Normal tissue		28%	34%	30%	43%	20%	

Asterisks indicate hypermethylation (>2SD from median).

In analysis of BRAF mutation status, 30/42 (73.1%) of analysed serrated adenomas were BRAF mutant, with 29/41 being V600E and 1/41 being V600L, in line with previous studies. Of the three TSA within the SA group, two had V600E mutations and the other was wild type and in the SSA group, 27/41 had BRAF mutations.

To study the relationship between methylation and expression in serrated adenomas, IHC for PCDH7 and SLIT2 was carried out on the 32 SAs within the verification group. For PCDH7 expression, 23/32 (72.0%) SA samples (23 SSA only) scored either a median expression level of 1 or 0 (minimal/no expression), and 9/32 (28.1%) had normal expression (6 SSA, 1 mixed TSA/SSA, 2 TSA), very similar to the control normal mucosa. For SLIT2, 31/32 (97%) SA samples (29/29 SSA, 1 mixed TSA/SSA and 1 TSA) had a median expression score of 1 or 0 (minimal/no expression), and 1/32 (3%) had normal expression (1 TSA). Inter-observer variation for PCDH7 expression was κ = 0.70 (SE = 0.12, Z = 5.60, p<0.001) and for SLIT2 expression κ = 0.74 (SE = 0.16,Z = 4.53, p<0.001). Of the SLIT2 samples, there was only 1 sample where there was disagreement between examiners, with examiner one scoring it as 2/3 and examiner two as 3/3. After discussion, the score for this sample was set at 3/3.

**Figure 4:**
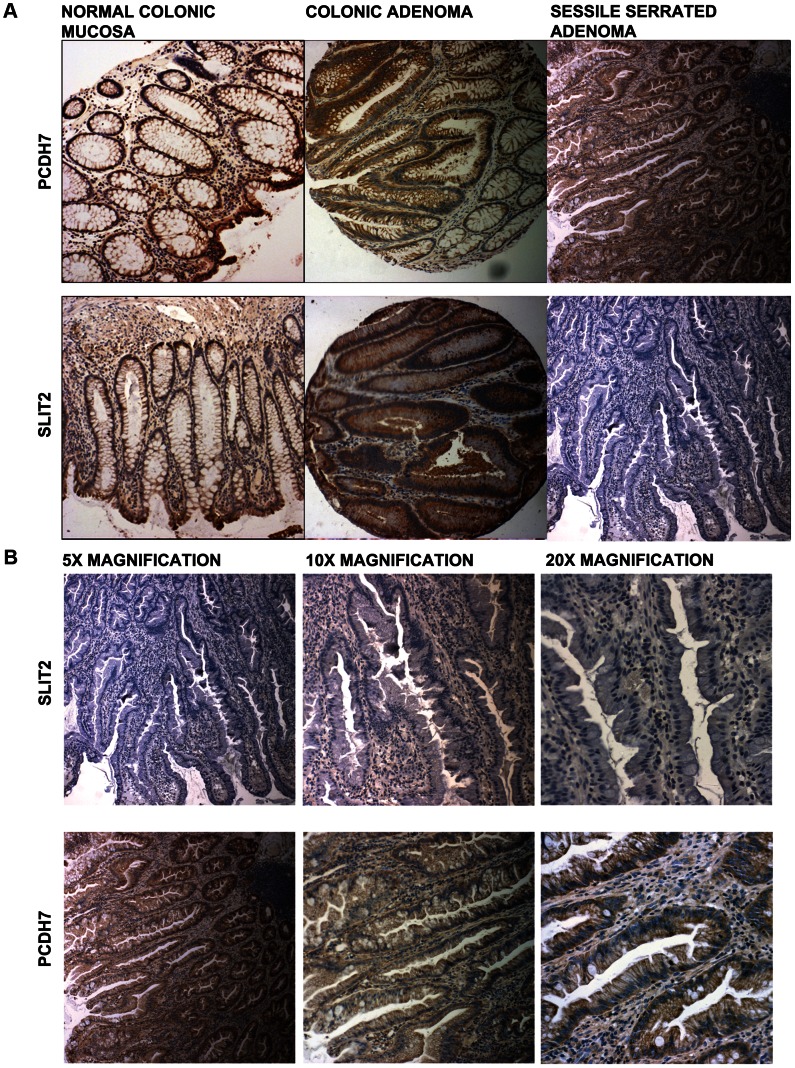
Photomicrographs. (a) Photomicrographs of IHC staining for PCDH7 and SLIT2 in normal colonic mucosa, colonic adenoma and serrated adenoma tissue. Expression of both PCDH7 and SLIT2 are shown by brown staining in the image. (b) 5×, 10× and 20× photomicrographs of SLIT2 & PCDH7 expression in serrated adenomas. Expression of both PCDH7 and SLIT2 are shown by brown staining in the image.

**Figure 5:**
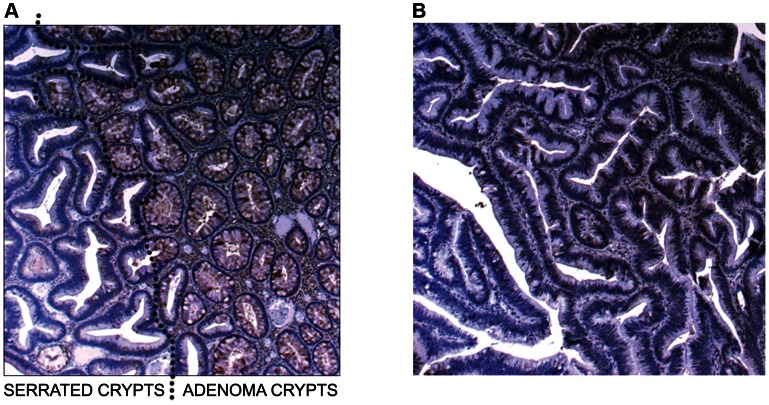
Image of IHC stains for SLIT2. (a) Image of IHC stain for SLIT2 on mixed serrated/adenomatous polyp. Dotted line shows demarcation between serrated and adenomatous crypts. Top right of image shows normal expression (adenomatous area). Bottom left of image shows loss of expression (serrated area). (b) Image of IHC stain for SLIT2 on pure serrated area of mixed polyp to demonstrate serrated morphology.

In Figure 4a (5× magnification) & 4b (5×, 10×, 20× magnification), normal expression of SLIT2 can be seen in both the conventional adenoma and normal tissue, whereas there is complete loss of expression in the serrated adenoma. A particularly interesting result was found in a polyp with mixed adenomatous/serrated architecture, as demonstrated in Figure 5. In this image, there was very reduced expression of SLIT2 within the crypts with serrated morphology but normal expression of SLIT2 within the crypts with “classical” adenomatous morphology.

There was a poor correlation between *PCDH7* promoter methylation and expression (Table 3), in SA, normal and classical adenoma tissues (n = 47) suggesting that there may be no relationship between the two (Fisher's exact test, p = 0.478). By contrast, there was a clear positive association between *SLIT2* methylation and loss of expression (Fisher's exact test, p = 4.97×10^−9^, Table 3). Similarly, in comparing PCDH7 expression vs. LOH at AFM112XD4 (Table 4), in samples where there was loss of PCDH7 expression there was significantly higher rates of LOH at AFM112XD4 (Fishers exact p = 0.001). In comparing SLIT2 expression vs. LOH at AFM112XD4 (Table 4), in samples where there was loss of SLIT2 expression there was significantly higher rates of LOH at AFM112XD4 (Fishers exact p = 5.16×10-9).

**Table 3:** Expression of PCDH7 and SLIT2 compared with methylation of PCDH7 and SLIT2.

	SSA (29 samples)		Mixed SSA/TSA (1 sample)		TSA (2 samples)		Classical adenomas (10 samples)		Normal tissue (5 samples)		
	PCDH7 methylation										
	Meth	Unmeth	Meth	Unmeth	Meth	Unmeth	Meth	Unmeth	Meth	Unmeth	Total
**No PCDH7 expression**	12	11	0	0	0	0	0	0	0	0	23
**PCDH7 expression**	0	6	0	1	0	2	0	10	0	5	24
**Total**	12	17	0	1	0	2	0	10	0	5	47

	SLIT2 methylation										
**No SLIT2 expression**	29	0	1	0	0	1	0	0	0	0	31
**SLIT2 expression**	0	0	0	0	0	1	0	10	0	5	16
**Total**	29	0	1	0	0	2	0	10	0	5	47

PCDH7 expression and methylation correlation: Fisher's exact p = 0.289.

Expression of SLIT2 compared with methylation of SLIT2 in 47 samples (32 SA, 10 normal mucosa samples, 5 classical adenomas): Fisher's exact p = 4.97×10^−9^.

**Table 4:** Expression of PCDH7 and SLIT2 compared with loss of heterozygosity at AFM112XD4.

	SSA (29 samples)		Mixed SSA/TSA (1 sample)		TSA (2 samples)		Classical adenomas (10 samples)		Normal tissue (5 samples)		
	AFM112XD4 LoH										
	Meth	Unmeth	Meth	Unmeth	Meth	Unmeth	Meth	Unmeth	Meth	Unmeth	Total
**No PCDH7 expression**	22	1	0	0	0	0	0	0	0	0	23
**PCDH7 expression**	4	2	1	0	0	2	0	10	0	5	24
**Total**	26	3	1	0	0	2	0	10	0	5	47

	AFM112XD4 LoH										
**No SLIT2 expression**	26	3	1	0	0	1	0	0	0	0	31
**SLIT2 expression**	0	0	0	0	0	1	0	10	0	5	16
**Total**	26	3	1	0	0	2	0	10	0	5	47

Correlation between loss of heterozygosity for AFM112XD4 and PCDH7 expression: Fishers exact p = 0.001.

Correlation between expression of SLIT2 and loss of heterozygosity at AFM112XD4 (32 SA, 10 normal mucosa samples, 5 classical adenomas: Fishers exact p = 5.16×10^−9^.

For the classical adenomas on the tissue microarray, 83/85 (97.6%) demonstrated strong expression of PCDH7, and 2/85 (2.4%) loss of expression. For SLIT2, 4/83 (4.8%) had a median expression score of either 1 or 0 (minimal/no expression) and 79/83 (95.2%) had normal expression.

As part of a separate independent study [13] conducted in our laboratory into whole genome methylation patterns comparing paired colorectal tumour and normal mucosa a gene set enrichment analysis (GSEA) was carried out comparing tumour vs. normal mucosa. On leading edge analysis (an analysis designed to ascertain the most frequently differentially methylated genes within a gene set) of the GSEA, SLIT2 was the most highly ranked gene in the set. This suggests SLIT2 may be implicated as a tumour suppressor in colorectal carcinogenesis.

## Discussion

A consistent and specific region of LOH occurs in both sessile serrated adenomas (irrespective of presence/absence of dysplasia), located around chromosome 4p15.1–4p15.31 (Chr 4:18,500,000–34,000,000). Of two excellent candidate genes within this region, *SLIT2* shows very frequent promoter methylation and low protein expression. The other gene, *PCDH7*, is not infrequently methylated, but shows no convincing evidence of reduced protein expression, and the association between methylation and expression is weak. It is conceivable that long range methylation may be occurring within this region, causing promoter methylation in a number of genes. However, there is no concordance between *SLIT2* & *PCDH7* methylation and so we thought that this was unlikely. A potential weakness of our study is the use of methylation specific PCR in analysing promoter methylation. As discussed in the methods, we felt that MSP provided a reasonable balance between cost and sensitivity, and we took all possible methods to reduce false positives. We verified the MSP using bisulfite pyrosequencing in a limited sample set, finding informative methylation within the *SLIT2* region studied. We were also reassured by the lack of methylation observed in both normal mucosa and classical adenoma samples, and the fact there was no long range methylation of *SLIT2* outside the identified region.

Alterations in the chromosome 4p15 region have been identified in a variety of cancers. Shivapurkar et al [14] demonstrated a LOH rate of 47% in 4p15 using microsatellite markers in a panel of colorectal cancers. In a separate study [15], they also demonstrated LoH in this region in 57% of breast cancers studied. This finding was also replicated [16] in a study of malignant mesotheliomas and small cell lung cancer (>50%). However, these findings may have been by aneuploidy that is commonly found in these types of cancer, whereas all our SAs were near-diploid as assessed by SNP arrays.

*SLIT2* has been postulated for several years to act as a tumour suppressor gene in several cancers [17]. On the basis that *SLIT2* expression is regulated by methylation, the findings of our experiment agree with those of Dallol et al [18]. They examined the role of *SLIT2* as a tumour suppressor in a panel of 32 colorectal cancers, finding that 23 (72%) were methylated in this region. Furthermore, after treatment of 6 colorectal cancer cell lines with the demethylation agent 5-aza-2-deoxycytidine (5-aza-C), they demonstrated re-expression of *SLIT2*. In order to ascertain whether *SLIT2* acted as a tumour suppressor, Dallol et al exposed colorectal cancer cell lines to ectopic SLIT2, finding that it could suppress colony formation.

The mechanism by which *SLIT2* exerts pro-apoptotic and growth-suppressive effects is unclear. Prasad et al [19] suggested that SLIT2 regulates the β-catenin and PI3K signalling pathways and enhances β-catenin/E-cadherin-mediated cell adhesion. The pro-apoptotic role of SLIT2 may be caused by interaction with the DCC pathway, as SLIT2 interacts with ROBO [20] which in turn interacts with DCC (20), with apoptosis occurring via the caspase pathway [21]. A recent study [22] has also identified low frequency SLIT2 methylation as a marker of high risk dysplasia in ulcerative colitis cancers.

This study is the first to demonstrate SLIT2 LOH, promoter methylation and down-regulation consistently occurring in a pre-malignant lesion (the sessile serrated adenoma), and may explain partially the alternate pathway by which sessile serrated adenomas progress towards carcinoma. We did observe a single TSA where there was loss of SLIT2 expression without LOH or SLIT2 promoter methylation. This could be because of somatic mutation within SLIT2, although this was not examined due to the excessive cost of sequencing SLIT2. Whilst further studies are required to show a causal relationship between loss of SLIT2 expression and the pathogenesis of SAs, in principle, the two hits we have found at *SLIT2* – methylation and LOH – could promote tumorigenesis by a revised version of the Knudson two hit hypothesis [23], in which LOH renders an epimutation homozygous.

## Materials and Methods

Forty-two formalin-fixed, paraffin-embedded (FFPE) serrated adenomas were obtained from Mayday University Hospital, Gloucester NHS Hospitals Foundation Trust and St George's Hospital, London NHS Trust. All patients had had colonoscopy for screening purposes and none reported a family history of colorectal cancer. Representative haematoxylin and eosin-stained sections were reviewed independently by two consultant histopathologists to confirm serrated architecture and the presence/absence of dysplasia. 100 “classical” adenomas from patients undergoing screening colonoscopy, but without a family history of cancer, were examined using a tissue microarray. Normal tissue samples were obtained from 10 unrelated patients. All mucosa samples sample from patients undergoing screening colonoscopy for a change in bowel habit who did not have a family history of cancer, no polyps on colonoscopy and on biopsy were found to have completely normal histological appearances, without any mucosal inflammation being present. Ethical approval was obtained from Wandsworth Ethics Committee (07/H0806/109) and Oxford Research Ethics Committee (05/Q1605/66). 40 µM tissue scrolls were cut and DNA extraction was performed using proteinase K digestion at 55°C. DNA was diluted to a concentration of 50 ng/µl.

Genome-wide SNP genotyping was carried out using the Illumina Goldengate HumanLinkage system (Panel V) on 10 serrated adenoma samples of which 9/10 were sessile serrated adenomas and 1/10 was a traditional serrated adenoma (TSA). This was included to see if the SSA and TSA demonstrated similar characteristics. Briefly, 1 µg of genomic DNA was amplified as per the manufacturer's instructions and hybridised to the Sentrix BeadArray overnight. The chip was then washed and stained, and scanned immediately using an Illumina BeadArray reader. GenomeStudio software which was used to identify regions with loss of heterozygosity (LOH) and/or copy number changes in unpaired sample mode DNA.

In order to verify the Illumina array results, 100 ng DNA from two sessile serrated adenoma samples were analysed using the Affymetrix OncoScan FFPE Express service utilising Affymetrix whole genome molecular inversion probe (MIP) SNP arrays. Data were analysed using GoldenHelix SNP & Variation Suite 7 utilising the copy number analysis function.

Microsatellites (D4S230, D4S418, D4S914, D4S616, D4S1593, D4S2289 and AFM112XD4) were chosen to cover the region of interest comprising chromosome bands 4p15.1–4p15.31 (Chr4:18,500,000–35,500,000, Genome Build 36). PCR products from the SAs (Text S1) underwent capillary electrophoresis fragment analysis on an Applied Biosystems 3730xl analyser. In the absence of paired normal DNA, we exploited the fact that the SA samples contained a small amount of contaminating normal cells and hence did not show complete LOH. On this basis, we were able to analyse unpaired samples of normal colonic mucosa with the same genotypes as the tumours for scoring LOH. In order to verify the levels of heterozygosity in our study against previously observed population norms for microsatellite markers, we also carried out microsatellite PCR for all analysed microsatellites on 96 blood derived DNA controls from the CORGI study (primers and conditions available in Text S1). Data were analysed using Applied Biosystems GeneMapper 4.0 software. Samples were called as having LOH if the peak area ratio of the second allele compared to the first was less than 0.5 or greater than 2.0.

For methylation-specific PCR (MS-PCR) & pyrosequencing, 1 µg genomic DNA was bisulphite converted using the Zymo DNA Methylation Gold bisulphite conversion kit and eluted into a 20 µL volume, which was then diluted to give a final concentration of 20 ng/µl. MS-PCR primers (conditions in Text S1) were designed using MethPrimer to span the CpG Island of the *PCDH7* promoter and the *SLIT2* promoter region [24] (primers and conditions available in Text S1). After MS-PCR, SA sample reaction products were run on a 2% agarose gel alongside those from unpaired normal colonic mucosa DNA, 100% methylated DNA and 100% unmethylated DNA as controls. Samples were classed as methylated if a band was visualised in the methylation-specific reaction and unmethylated if no band was present in the methylated reaction but was present in the unmethylated reaction. If there was a band in both samples, the sample was said to be methylated as this could represent either hemi-methylation or heterogeneity for methylation within the sample. If there were no bands in either sample, the sample was said to have failed. It was thought that MSP would provide an adequate method due to use of highly sensitive and specific PCR reagents (Qiagen Multiplex PCR kit), the low number of cycles used in the PCR reaction (35 cycles) and previous studies documenting the reasonable sensitivity and specificity of MSP [25]–[27]. However, to verify the results, bisulfite pyrosequencing primers (primer sequences available in Text S1) were designed to sequence the region of interest using Pyromark Assay Design v2.0 (Qiagen). A off-the-shelf primer set for the SLIT2 CpG island (PM00018634), available from Qiagen, was also used to analyse long range methylation of *SLIT2*. A bisulfite pyrosequencing PCR was set up with standard conditions (available in Text S1) and carried out on a limited subset of samples. Products were analysed using a PyroMark Q96 instrument.

To analyse *BRAF* mutation status, 20 ng of SA DNA was used in a standard sequencing PCR reaction for the region surrounding BRAF codon 600, and sequenced using an ABI BigDye sequencing reaction (primers & conditions available in Text S1).

4 µM serrated adenoma sections were cut from FFPE blocks and mounted on poly-L-lysine glass slides for use in immunohistochemistry. Immunohistochemistry for PCDH7 was performed using an indirect method against the primary antibody (Sigma Prestige rabbit anti-PCDH7 (ID HPA011866), 1∶100 dilution or Sigma Prestige rabbit anti-SLIT2 (ID HPA023088, 1∶35 dilution) for 1 hr. A positive control of normal colonic mucosa obtained from a normal screening colonoscopy of an individual without colorectal tumours or a family history of colorectal cancer was used, and the negative control was an SA which followed all staining steps except application of the primary antibody. Additional comparison was made with 100 classical colonic adenomas was made using an in-house tissue microarray and with 5 normal colonic tissue samples.

PCDH7 and SLIT2 IHC slides were read by two independent assessors (see Acknowledgements). From normal tissue in this study, expression of both PCDH7 and SLIT2 was found to be primarily cytoplasmic, as previously observed by the Human Protein Atlas project. IHC scoring was graded from 0–3 (0 = no expression, 1 = mild expression, 2 = moderate expression, 3 = heavy expression) as compared to the normal control (heavy expression) and the median of the two observers was calculated. If there was any disagreement, the higher expression value was used, as this was conservative, based on an assumption that loss of expression would be functionally important. Assessment of inter-observer variation was estimated using Cohen's Kappa (κ).

As part of a separate independent experiment [13], paired colorectal tumour and normal mucosa was also analysed for whole genome methylation patterns on the Illumina HumanMethylation27 platform and subjected to analysis via Gene Set Enrichment Analysis(GSEA).

Statistical analyses were carried out using STATA 11.1 (StataCORP, Texas). For the SLIT2 and PCDH7 protein expression, variables were dichotomised into strong/moderate expression (2 or 3), or no/weak expression (0 or 1). For all comparisons, we used the χ^2^ test, or Fisher's exact test if any cell within the table had a count of less than five.

## Supporting Information

Text S1 Protocols for SLIT2 MSP, SLIT2 pyrosequencing, BRAF sequencing and 4p15 microsatellite PCR.
(DOCX)

## References

[1] Leggett B, Whitehall V Role of the serrated pathway in colorectal cancer pathogenesis. Gastroenterology 138: 2088–2100. 20420948 10.1053/j.gastro.2009.12.066

[2] Snover DC, Jass JR, Fenoglio-Preiser C, Batts KP (2005) Serrated polyps of the large intestine: a morphologic and molecular review of an evolving concept. Am J Clin Pathol 124: 380–391. 16191506 10.1309/V2EP-TPLJ-RB3F-GHJL

[3] Rex DK, Ahnen DJ, Baron JA, Batts KP, Burke CA, et al (2012) Serrated Lesions of the Colorectum: Review and Recommendations From an Expert Panel. Am J Gastroenterol 10.1038/ajg.2012.161 PMC3629844 22710576

[4] Torlakovic EE, Gomez JD, Driman DK, Parfitt JR, Wang C, et al (2008) Sessile serrated adenoma (SSA) vs. traditional serrated adenoma (TSA). Am J Surg Pathol 32: 21–29. 18162766 10.1097/PAS.0b013e318157f002

[5] Carr NJ, Mahajan H, Tan KL, Hawkins NJ, Ward RL (2009) Serrated and non-serrated polyps of the colorectum: their prevalence in an unselected case series and correlation of BRAF mutation analysis with the diagnosis of sessile serrated adenoma. J Clin Pathol 62: 516–518. 19126563 10.1136/jcp.2008.061960

[6] Chan TL, Zhao W, Leung SY, Yuen ST (2003) BRAF and KRAS mutations in colorectal hyperplastic polyps and serrated adenomas. Cancer Res 63: 4878–4881. 12941809

[7] Jass JR, Baker K, Zlobec I, Higuchi T, Barker M, et al (2006) Advanced colorectal polyps with the molecular and morphological features of serrated polyps and adenomas: concept of a ‘fusion’ pathway to colorectal cancer. Histopathology 49: 121–131. 16879389 10.1111/j.1365-2559.2006.02466.x PMC1619718

[8] O'Brien MJ, Yang S, Mack C, Xu H, Huang CS, et al (2006) Comparison of microsatellite instability, CpG island methylation phenotype, BRAF and KRAS status in serrated polyps and traditional adenomas indicates separate pathways to distinct colorectal carcinoma end points. Am J Surg Pathol 30: 1491–1501. 17122504 10.1097/01.pas.0000213313.36306.85

[9] Vandrovcova J, Lagerstedt-Robinsson K, Pahlman L, Lindblom A (2006) Somatic BRAF-V600E mutations in familial colorectal cancer. Cancer Epidemiol Biomarkers Prev 15: 2270–2273. 17119056 10.1158/1055-9965.EPI-06-0359

[10] Fu B, Yachida S, Morgan R, Zhong Y, Montgomery EA, et al (2012) Clinicopathologic and genetic characterization of traditional serrated adenomas of the colon. Am J Clin Pathol 138: 356–366. 22912351 10.1309/AJCPVT7LC4CRPZSK PMC3556914

[11] Kim GE, Lee KH, Choi YD, Lee JS, Lee JH, et al (2011) Detection of Slit2 promoter hypermethylation in tissue and serum samples from breast cancer patients. Virchows Arch 459: 383–390. 21894562 10.1007/s00428-011-1143-5

[12] Zhou WJ, Geng ZH, Chi S, Zhang W, Niu XF, et al (2011) Slit-Robo signaling induces malignant transformation through Hakai-mediated E-cadherin degradation during colorectal epithelial cell carcinogenesis. Cell Res 21: 609–626. 21283129 10.1038/cr.2011.17 PMC3203654

[13] Beggs AD, Jones A, El-Bahwary M, Abulafi M, Hodgson SV, et al (2012) Whole-genome methylation analysis of benign and malignant colorectal tumours. J Pathol 10.1002/path.4132 PMC3619233 23096130

[14] Shivapurkar N, Maitra A, Milchgrub S, Gazdar AF (2001) Deletions of chromosome 4 occur early during the pathogenesis of colorectal carcinoma. Hum Pathol 32: 169–177. 11230704 10.1053/hupa.2001.21560

[15] Shivapurkar N, Sood S, Wistuba, II, Virmani AK, Maitra A, et al (1999) Multiple regions of chromosome 4 demonstrating allelic losses in breast carcinomas. Cancer Res 59: 3576–3580. 10446964

[16] Shivapurkar N, Virmani AK, Wistuba II, Milchgrub S, Mackay B, et al (1999) Deletions of chromosome 4 at multiple sites are frequent in malignant mesothelioma and small cell lung carcinoma. Clin Cancer Res 5: 17–23. 9918198

[17] Dallol A, Da Silva NF, Viacava P, Minna JD, Bieche I, et al (2002) SLIT2, a human homologue of the Drosophila Slit2 gene, has tumor suppressor activity and is frequently inactivated in lung and breast cancers. Cancer Res 62: 5874–5880. 12384551

[18] Dallol A, Morton D, Maher ER, Latif F (2003) SLIT2 axon guidance molecule is frequently inactivated in colorectal cancer and suppresses growth of colorectal carcinoma cells. Cancer Res 63: 1054–1058. 12615722

[19] Prasad A, Paruchuri V, Preet A, Latif F, Ganju RK (2008) Slit-2 induces a tumor-suppressive effect by regulating beta-catenin in breast cancer cells. J Biol Chem 283: 26624–26633. 18611862 10.1074/jbc.M800679200 PMC2546548

[20] Prasad A, Qamri Z, Wu J, Ganju RK (2007) Slit-2/Robo-1 modulates the CXCL12/CXCR4-induced chemotaxis of T cells. J Leukoc Biol 82: 465–476. 17565045 10.1189/jlb.1106678 PMC2286829

[21] Forcet C, Ye X, Granger L, Corset V, Shin H, et al (2001) The dependence receptor DCC (deleted in colorectal cancer) defines an alternative mechanism for caspase activation. Proc Natl Acad Sci U S A 98: 3416–3421. 11248093 10.1073/pnas.051378298 PMC30668

[22] Azuara D, Rodriguez-Moranta F, de Oca J, Sanjuan X, Guardiola J, et al (2012) Novel methylation panel for the early detection of neoplasia in high-risk ulcerative colitis and Crohn's colitis patients. Inflamm Bowel Dis 10.1002/ibd.22994 22532293

[23] Jones PA, Laird PW (1999) Cancer epigenetics comes of age. Nat Genet 21: 163–167. 9988266 10.1038/5947

[24] Li LC, Dahiya R (2002) MethPrimer: designing primers for methylation PCRs. Bioinformatics 18: 1427–1431. 12424112 10.1093/bioinformatics/18.11.1427

[25] Hall GL, Shaw RJ, Field EA, Rogers SN, Sutton DN, et al (2008) p16 Promoter methylation is a potential predictor of malignant transformation in oral epithelial dysplasia. Cancer Epidemiol Biomarkers Prev 17: 2174–2179. 18708411 10.1158/1055-9965.EPI-07-2867

[26] Parrella P, la Torre A, Copetti M, Valori VM, Barbano R, et al (2009) High specificity of quantitative methylation-specific PCR analysis for MGMT promoter hypermethylation detection in gliomas. J Biomed Biotechnol 2009: 531692. 19503806 10.1155/2009/531692 PMC2688744

[27] Shaw RJ, Akufo-Tetteh EK, Risk JM, Field JK, Liloglou T (2006) Methylation enrichment pyrosequencing: combining the specificity of MSP with validation by pyrosequencing. Nucleic Acids Res 34: e78. 16807314 10.1093/nar/gkl424 PMC1904102

