# Supplementary material for: Loss of Expression and Promoter Methylation of SLIT2 Are Associated with Sessile Serrated Adenoma Formation
Source: PLoS Genet. 2013 May 9;9(5):e1003488. doi: 10.1371/journal.pgen.1003488 (PMC3649993; doi:10.1371/journal.pgen.1003488)
Supplement: Text S1 — Protocols for SLIT2 MSP, SLIT2 pyrosequencing, BRAF sequencing and 4p15 microsatellite PCR. (DOCX) [file pgen.1003488.s001.docx]

**Supplementary methods S1**

**SLIT2 MSP**

Input sequence – CpG island 291 5’ to SLIT2.

>hg18_cpgIslandExt_CpG: 291 range=chr4:19862375-19865966 5'pad=0 3'pad=0 strand=+ repeatMasking=none

***Yellow shading highlights input sequence to MethPrimer. Red text highlights location of MSP primers.***

CGGGTGGCTCCTTCTTCGGGCCAAGTTTGCCCGCGCGTGGGAGCGAGGGGGCGAGCCAGGGCGCACGGGAACGCGCGCCGCCAGCCGCCGCCTC

CGCCCAGCGCTGGCAGCCCCGGCCTCACCTCTCCCCTCTCCCCAGCGAGGTGCGGGCTCCCAGAGCGGAGCCAGGGGGCGGGCCTGGGCCTCGA

GGGGCGGGGTCAGGGGGCGGAGGCTCGGGCGGCTATATAAGGCCCGCGCCGGCTCAACTTCGGACTTGGTGTTATTTATTTGGGAAGCGCCCGG

ACGGCGGAGCTTGGCGGCGGCGGTGGTGGTGGCTGCCGCAGACTGTGGTTAAAAAAAAGAAGGCGGCGGCGGCGGCGGCGGCGGAGGCGGAGGC

AGCTGCGAGGCATGGGAGCGCCGAAGCGCCCAGGCGCAGGCCGAAGCTGCCGCGCTTTCTGGGCACGGCGGGAGTGCTGAGCAGAAAGGGGAGC

GCCGGGGGCCCGCAGCCGGCTCCGGAGGCGCGGGCCGGGTTTTTGTTTGGCTACGCTGAGCGCCAGTCAGCCCCAGCGAACAACTCCAGTTACG

ACAACAACCCACCTTCCTTCCAGACAAGCGAGACTTGGGCTGCTGCGTCCGTCCTATTGTTTAGACACTTGCCAGGGGCTCCGGAGTCGGCAGA

GCCACCGAGTCCCCGCTCTGAGTCGTCGCCCTCCCTCTCCCCGACCTCGCTCCCTGGAGCGGGAGGCCAGGAAAGCAGCGGGAGAGGGGAAGGG

GCTAGAAGGAGAAGGACTACCCGGGACTGCGGCCGCCGCGTCAGGTGCAGCGCCAGGAGCCGGGCGGCGTCGCCACGCCGGCAGGGGTACCGCC

ACTGTGGCCTTGGGGGACGGAATTCAAAGCCTGGGAAAAGTTGCTGCACTTTGAGAAGGACGAACCACTAGTGGGAGACCGCCGGGGGCCGGCC

GTGGCTCTGCGCCCTCCGGAACCCGGCTCTTGTTTCTTCTACCTTTGCCATCAGGTGTCTGCCGCGGAGCTGCGGCTTATCTGGGAGACGAGCG

GGGTTGACACGCGCGCACACACTACTGCCATTCAGCTGCCGCCTGGCTCTGCCTGGAGTAGTGGATCCCACCCGCCCACCTGCCACCGAGCCAT

TCTCCAGTACGCCCCAGCAGGACGCTGACACCTCCAACCTTGGCCTTTGCCTTTCCACTCCTTCCGGTCTGCCTGGTTTTTAAGTCCGCCCCCA

GTCAGTCCCCACTCAGTCTTCGCAGCAGCTCTCATCCTCCACTTGGCCTCTTGGAGTTCCTCGCCGGAGTGCTGACTAGTGGATATTTCTGCCC

GGCTGCGGCGGCCCGACTGCCCTTTTGTCTTTTCTGCGTGACCTCGGGGCAGGTCCTGGTGCAGAGCGTCGCCAAGGACGCCGAGCGGGAGGCG

GGATTGCCCAGACATCCTTCAGCGAAGTGCATGTGTGTTTGTAAACCATCGTTGGCTGTCGGGAGACCGCGAGGACCGGTCCAGGCTGCGGCGG

AGTCGAGGGCGAGGGAGAGGCCGCGTGAGTGAGCAGAGTCCAGAGCCGTGCGCCCCCAGAACTGCGCGTCCGCCCCGTGCACCCCCGCGCGCCA

TGCCCAGTTGCCCCGCGCGCTCTGCTACGGGCCCGCTGGGCTTCCGCGCCTTCTAGCTTCCGGAGCCCACTTTGATCGGGGCCATAATACCTAT

TGAGATCCCCTCTTCTGTCTTGTACCTTCGCCACTGGCATCGGATTTGCAGAAGCGTGCGTGGGATCAGAGGACCGCCCTCCCCACAACAACCG

GCCCCTGCATCTTAGCAGCCGTTGGAAGCCCCAGCTCTTTTACCGCCAAGTTCATCCTTGGGAGACAGAAGACGCGTGATCTCCTCTCCGCTGC

TCTTGGGGTCTCCTTGCAGCCCTGGCCAGGCGGATTCATCCTCAGGACCTAAAGTTGCCCAAGGAGCTCCTGCTCTGCCAGAGGAGGGTGGAGA

GGGCGGTGGGAGGCGTGTGCCTGAGTGGGCTCTACTGCCTTGTTCCATATTATTTGGTGCACATTTTCCCTGGCACTCTGGGTTGCTAGCCCCG

CCGGGCACTGGGCCTCAGACACTGCGCGGTTCCCTCGGAGCAGCAAGCTAAAGAAAGCCCCCAGTGCCGGCGAGGAAGGAGGCGGCGGGGAAAG

ATGCGCGGCGTTGGCTGGCAGATGCTGTCCCTGTCGCTGGGGTTAGTGCTGGCGATCCTGAACAAGGTGGCACCGCAGGCGTGCCCGGCGCAGT

GCTCTTGCTCGGGCAGCACAGTGGACTGTCACGGGCTGGCGCTGCGCAGCGTGCCCAGGAATATCCCCCGCAACACCGAGAGACTGTGAGTATG

CGCTCTTCGTCTTCCCCTCTCCCCATCCGGGCCGCGCACCCCTGCCTCCACTGGAGGAACCTGTCAGCTCAGGGTCCTGTGCCTGGGGCAGCCC

TCGCTAGCTCTCCCCCATGCACATCCTGGGGTTGAGCTCTCCGGGAGGGCACTGGCCAGGGAAGGGCCTCTGTCCAAGGAGGGGCGGGTCCGCT

GGCAGCTGCGCTAGTTCTCCCTCCCCTGCTCTCGTCCCGCCACTCGCAGCTCCTTGCTGGCTAGTTCTCTGGGGCTGGGGAGCGGGTAGATAGG

GGACAAGTACTGGAGGATGCCCGGGGCAAGTGAGACGCCACTTTGTTCTCCAGAGTCCATAAACGGAGTCACCTTGCGATTGCCAGCATCCAGG

TCGGTTTCAGAGCCCAGTCCTCGCTCTTGTCGCAGGCTGGCGCGGAGGGGATAGCAGGGAGACTCAAAAGAGAGAAACTTGCCTTCCCCGATTT

TTTGTCACCCTCCTGGGGGCGAAGGTTAGGAAGAAGGGGTCATGGAGTGCCTGGGGGTGCTTCTCACAGGTCGCGGGGAGAAGGGTGCCCCAGG

ACGGCGACACCTCGCATAGTAGCCTCGCGCAGCCCCCCGCCCCCCACTTCTCCGGGGAGGGGAAGACGGCGTCAGGCCCCTAGGGACTTGTCTC

AGCGGGCGACTGCGAGGGAGGACCGTGTCCCATCCGTTAAGCGAAGTTAGCACTGGTTCTCCAGCGCAAACCAGCCCAACCAGGTCTTACCACT

GCGGCGACCCGGCGGTGCCCGGCTGCCCCCTCCGGCCCTTCCTGCTGAACCCCTGCGTCCCCATCCACCTTTCTGGCAGTTTCTGCGCCCCTTC

ACGTGGCAGCAGTTCCCCTGCCTTCCCCTCTTCGCGCTCCGTTGCTCGCAGACGTCCCCGCCTCCCTGTCTTTGCGAGTCTCTAATGAAGAAGT

AAATGCAGACCCGGTGTTGACGGCCCACGCGCTCCTGATGAGGCGCTTCCAGAGTTCAGCGAAGTGGAGCATGGAGGCCGTTCTCTTTGTGAAC

GCCGAGGCCGCCGCCCCGCGCCAGTCCGGCCGCCCTAGGCACGTTCCGCTCTCGCGGTTGCGTGTGGGCCGGGAGTAAGCGAAGGGCGCGGGGC

TAGGCCGGCAGGGGCCAGCGCGTCTGGATTCGTCCCGCGGCCTCTTTGCCCCCGGGGTGTAAGGGTTTCGGGCCGTGGAGAGGAGGCGCTCTGA

CCCGCACGTCTCCACCCGCG

**Methprimer Output**

Sequence Name: SLIT2 MSP

Sequence Length: 450

CpG island prediction results

Criteria used: Island size > 100, GC Percent > 50.0, Obs/Exp > 0.60

1 CpG island(s) were found in your sequence

Size (Start - End)

Island 1 296 bp (100 - 395)

Primer picking results for methylation specific PCR (MSP)

Primer Start Size Tm GC% 'C's Sequence

Left M primer 242 25 58.69 60.00 7 CGGGATTGTTTAGATATTTTTTAGC

Right M primer 380 24 59.67 66.67 4 CTAAACTCTACTCACTCACGCGAC

Product size: 139, Tm: 65.9

Left U primer 240 27 58.47 62.96 7 GGTGGGATTGTTTAGATATTTTTTAGT

Right U primer 381 25 54.70 64.00 4 TCTAAACTCTACTCACTCACACAAC

Product size: 142, Tm: 64.3

**Reaction MSP conditions:**

Use Qiagen Multiplex Taq (Multiplex Kit)

Master Mix 7.5uL

Forward primer (0.2uM) 0.3uL

Reverse primer (0.2uM) 0.3uL

Q solution 3uL

Water 1.9uL

DNA 20ng in 2uL

Annealing temperature – 56^o^C

**SLIT2 pyrosequencing**

| **Pyrosequencing Assay Design Analysis Report** |
| --- |

| **Assay Name** | SLIT2 covering MSP region |  |  |
| --- | --- | --- | --- |
| **Assay Type** | Methylation Analysis (CpG) |  |  |
| **Direction** | Forward |  |  |
| **Description** | SLIT2 Pyro covering MSP region | | |
| **Notes** |  | | |

| **Primer Set 1** | | | **Score: 76 Quality: Medium** | | |  |
| --- | --- | --- | --- | --- | --- | --- |
| **Primer** | **Id** | **Sequence** | **Nt** | **Tm, ºC** | **%GC** |  |
| 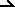PCR | F1 | GGGAGGAGGGATTGTTTAGATATT | 24 | 57.3 | 41.7 |  |
| 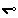PCR | R1 | CACCACTCTAAACTCTACTCACTCA | 25 | 53.9 | 44.0 |  |
| 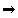Sequencing | S1 | AGGAGGGATTGTTTAGATATTTTTT | 25 | 46.5 | 28.0 |  |
| Target Polymorphisms | Position18, Position19, Position20, Position21, Position22, Position23, Position24, Position25, Position26, Position27 | | | | | |
| Sequence to Analyze | AGYGAAGTGT ATGTGTGTTT GTAAATTATY GTTGGTTGTY GGGAGATYGY GAGGATYGGT TTAGGTTGYG GYGGAGTYGA GGGYGAGGGA GAGGT | | | | |  |

**PCR reaction conditions:**

Annealing temperature 56^o^C

Used Qiagen PyroMark PCR kit

X1

Master Mix 12.5

CoralLoad buffer 2.5

Mg2+ 1.5

Q solution 5

Primer 2.5

DNA 1uL of 20ng/uL DNA

**BRAF sequencing**

For BRAF sequencing PCR, 1μL of purified crypt DNA was made up in a PCR reaction with 7.5μL of master mix (MM) from the Qiagen Multiplex PCR kit, 0.3 μL of BRAFV600E forward primer, 0.3 μL of BRAFV600E reverse primer, 3 μL of Q solution and 2.9 μL of ddH_2_O. The PCR reaction was carried out using an hot start step of 95^o^C for 15 minutes followed by 40 cycles of 95^o^C (denaturation) for 30 seconds, 58^o^C (annealing) for 1 minute 30 seconds and (extension) 72^o^C for 30 seconds, with a final extension step at 72^o^C for 10 minutes.

**BRAFV600E** Fwd : 5′-TGCTTGCTCTGATAGGAAAATG-3′

Rev : 5′-CCACAAAATGGATCCAGACA-3′

**Microsatellite PCR for 4p15 region:**

AFM112XD4 Fwd: 5’-[6FAM]TAATGAGTCATGGTGGAGGC-3’

Rev: 5’-CTATTCAAAACTGTCAAGATTTGCT-3’

D4S230 Fwd: 5’[HEX]TAGGAATAGGAAACAAATGCA-3’

Rev: 5’-TTAGGATGCTGACTTCACCA-3’

D4S418 Fwd: 5’-[6FAM]TTGGAGGTGGGGTCTAATG-3’

Rev: 5’-ATCACAGAGTGAAGNAGGATG-3’

D4S914 Fwd: 5’-[6FAM]GTGAGATGCTCAATATTCACATGG-3’

Rev: 5’-AGACAATGTAGGGAGTTCTGAGG-3’

D4S313 Fwd: 5’-[6FAM]CTCACATAAAAGCATCTACCGTTC-3’

Rev: 5’-CTGGAGAGGTAAGTGGCTGAAG-3’

D4S616 Fwd: 5’[6FAM]CAAATACTGTTACCCATGGTCACTT-3’

Rev: 5’-GACTCCAAGGTGAGCAGGTTTTC-3’

D4S1593 Fwd: 5’-[HEX]-ACCTTGTTGACTGGTTACTG-3’

Rev: 5’-AATTACATGAGGCAATTCC-3’

D4S2289 Fwd: 5’-[6FAM]-TTGGAATATCAGATGGAAAGG-3’

Rev: 5’-GCATGGCATTCCTATGACAC-3’
